# Prevalence of cardiac sarcoidosis in white population: a case–control study: Proposal for a novel risk index based on commonly available tests

**DOI:** 10.1097/MD.0000000000004518

**Published:** 2016-08-12

**Authors:** Magdalena M. Martusewicz-Boros, Piotr W. Boros, Elżbieta Wiatr, Jacek Zych, Dorota Piotrowska-Kownacka, Kazimierz Roszkowski-Śliż

**Affiliations:** a 3^rd^ Lung Diseases Department; b Lung Pathophysiology Department, National TB & Lung Diseases Research Institute; c 1^st^ Department of Clinical Radiology, Medical University of Warsaw, Warsaw, Poland.

**Keywords:** cardiac sarcoidosis, epidemiology, heart involvement, prevalence, risk factors, risk index, sarcoidosis

## Abstract

Cardiac sarcoidosis (CS) is a life-threatening and underdiagnosed manifestation of the disease, which requires a complicated and expensive diagnostic pathway. There is a need for simple tool for practitioners to determine the risk of CS without access to specialized equipment.

The aim of study was to determine the prevalence of CS in a group of patients diagnosed with or followed up because of sarcoidosis. A secondary objective was the search for factors associated with heart involvement.

We performed a prospective case–control study (screening analysis) in consecutive sarcoidosis patients collected from October 2012 to September 2015. Cardiac magnetic resonance (CMR) imaging was performed to confirm or exclude cardiac involvement in all patients. The study was conducted in a hospital-based referral center for patients with sarcoidosis and other interstitial lung diseases.

Analysis was performed in a group of 201 patients (all white) with biopsy-proven sarcoidosis, mean age 41.4 ± 10.2, 121 of them (60.2%) males. Four patients with previously recognized cardiac diseases, which make CMR imaging for CS inconclusive, were not included.

Cardiac involvement was detected by CMR in 49 patients (24.4%). Factors associated with an increased risk of CS (univariate analyses) included male sex (odds ratio [OR]: 2.5; 1.21–5.16, *P* = 0.01), cardiac-related symptoms (OR: 3.53; 1.81–6.89, *P* = 0.0002), extrathoracic sarcoidosis (OR: 3.48; 1.77–6.84, *P* = 0.0003), elevated serum NT-proBNP (OR: 3.82; 1.55–9.42, *P* = 0.004), any electrocardiography abnormality (OR: 5.38; 2.48–11.67, *P* = 0.0001), and contemporary radiological progression sarcoidosis in the lungs (OR: 2.98; 1.52–5.84, *P* = 0.001). Abnormalities in echocardiography and Holter ECG were also risk factors, but not significant in multivariate analyses. A CS Risk Index was developed using a multivariate model to predict CS, achieving an accuracy of 82%, sensitivity of 50%, specificity of 94%, and likelihood ratio 8.1.

CS was detected in one fourth of patients. A CS Risk Index based on the results of easily accessible tests is cost-effective and may help to identify patients who should be urgently referred for further diagnostic procedures.

## Abstract 2

Supplemental Digital Content is available in the text

## 1 Introduction

Sarcoidosis is a systemic granulomatous disease of unknown etiology predominantly affecting lungs and lymph nodes; however, any other organ may be involved.[ ^1^ ^2^] Sarcoidosis affects people of all races and ethnic groups and occurs at all ages. The prevalence is 10 to 80 cases per 100,000, depending on geographic and demographic variation.[ ^3^ ^4^] There are still no conclusive data regarding the etiology and the reasons for the differences in manifestation of the disease in different populations. [^5^ ^6^ ^7^ ^8^]

Cardiac involvement in sarcoidosis carries a poor prognosis.[ ^9^ ^10^] Sudden cardiac death owing to malignant ventricular tachyarrhythmias or advanced heart block can occur even in previously asymptomatic patients. The process of recovery, which includes fibrosis, may lead to overt heart failure. Cardiac sarcoidosis (CS) may occur at any stage of the disease, even in patients without typical changes in the respiratory tract or elsewhere.[ ^11^ ^12^] The prevalence of CS is not exactly recognized. Clinically evident CS has been noted in 2% to 7% of patients, although autopsy studies reported cardiac involvement in about one-fourth of cases in the United States or Europe and higher in Japan.[ ^9^ ^11^ ^13^] CS is a leading cause of sarcoid-attributed death (85% in Japan). Experts disagree on the criteria for optimal diagnosis and treatment. [^14^ ^15^ ^16^] However, early diagnosis of CS and early initiation and prolonged continuation of corticosteroid therapy seem to improve the prognosis. [^17^ ^18^ ^19^ ^20^] Therefore, there is a need for a simple tool for clinicians to predict CS. ^[16]^

The National TB & Lung Diseases Research Institute in Warsaw, Poland, serves as the national referral center for patients with sarcoidosis and other interstitial lung diseases, providing an opportunity to estimate the prevalence of active cardiac involvement in one of the much larger group of sarcoidosis patients, than previously described. [^21^ ^22^ ^23^ ^24^ ^25^]

### 1.1 Aim of study

The main purpose of the study was to evaluate the prevalence of active cardiac involvement in the group of patients diagnosed or followed up because of pulmonary sarcoidosis. A secondary objective was to find easily accessible predictors of CS.

## 2 Methods

We performed a case–control study for cardiac involvement in the group of prospectively collected consecutive patients with biopsy-proven sarcoidosis from October 2012 to September 2015 (36 months). We collected demographic information, medical history, including comorbidities, and medical treatment. We excluded patients with previously recognized cardiac diseases (including previously recognized CS), particularly coronary artery disease, which make cardiac magnetic resonance (CMR) imaging for CS inconclusive. Patients treated currently or in the past year for sarcoidosis (regardless of involved organ) were also excluded. All patients provided informed consent before study participation. All patients were directly asked about any complaints, a history of palpitation, presyncope and syncope, dyspnoea, deterioration in exercise, and nonspecific symptoms (like chest discomfort). Standardized evaluation of sarcoidosis patients included the following tests: serum activity of angiotensin-converting enzyme (ACE), N-terminal of the prohormone brain natriuretic peptide (NT-pro BNP) level, Troponin T STAT (Short Turn Around Time), calcium metabolism, radiological chest examination (assessed by experienced radiologists with comparison to previous examinations), standard 12-lead electrocardiography (ECG), pulmonary function tests (PFT), 6-minute walking test (6MWT), abdominal ultrasonography, ophthalmology assessment (or other specialist consultations, if needed), echocardiography (ECHO), and 24h-Holter monitoring.

All patients had a CMR study using a 1.5 T scanner (Siemens, Avanto SQ-engine T-class Tim [76x32]) equipped with a dedicated 32- channel cardiac coil. The protocol consisted of function, edema, and late gadolinium enhancement (LGE) imaging. Details of the method are provided in eMethods section (1 Supplemental Digital Content).

CMR findings were considered to be positive for active CS if both increased signal intensity on T2-weighted sequences (edema) and delayed contrast enhancement were present. X-ray coronary angiography was performed to exclude obstructive coronary artery disease (especially in cases with subendocardial pattern of enhancement). If the cause of myocarditis features in CMR study was ambiguous (possibly because of a viral infection), serological tests were performed. The final diagnosis for active myocarditis owing to CS disease was confirmed by a positive CMR test without evidence of other reasons for such abnormalities. ^[26]^ An example of a positive CMR study is shown in eFigure 1 (see in 2 Supplemental Digital Content). The study was approved by Ethic Committee in National TB & Lung Diseases Research Institute (KB-108/2012).

### 2.1 Statistical Analysis

Descriptive data were presented as mean ± standard deviation and range or 90% CI where indicated. Group comparisons were made using paired *t* tests for independent samples. The incidence ratios were presented as numbers of patients in groups and percentages. The Pearson *χ* ^2^ test was used to check for differences in the prevalence of observations. Univariate predictors for CS were identified. The diagnostic sensitivities and specificities, and the positive and negative predictive values of each baseline testing significant variable were calculated. Significant variables were included into multivariate analysis. Logistic regression analysis was used to build the model of association between risk factors and the outcome. Taking into account results of logistic regression analyses, we developed “Cardiac Sarcoidosis Risk Index” (CSRI). The required sample size for the comparison of the area under the receiver-operating characteristic (ROC) curve with a null hypothesis value was exceeded and the ROC curve was constructed. The method of DeLong et al (1988) for the calculation of the standard error of the area under the curve was used to assess the parameters of the developed index. The sensitivities and specificities, and the likelihood ratios for every CSRI value compared to results of CMR (as a “criterion standard”) for the diagnosis of CS in the cohort, were calculated. All statistical analyses were performed using STATISTICA (data analysis software system StatSoft, Inc. 2010), version 9.1 and MedCalc Statistical Software version 15.8 (MedCalc Software bvba, Ostend, Belgium; https://www.medcalc.org; 2015).

## 3 Results

During the 3-year study period, 201 consecutive sarcoidosis patients, all white, were included (Table 1). In more than half of them CMR study was performed in the first 6 months from the onset of the disease.

**Table 1:**
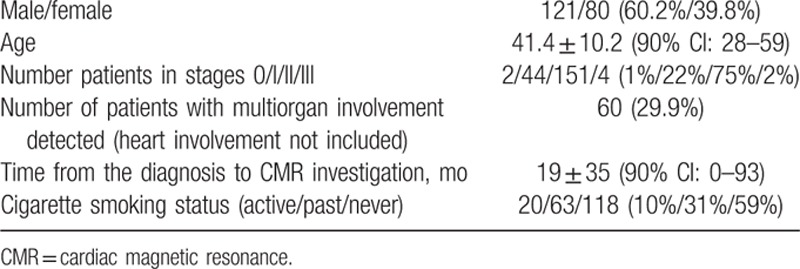
Characteristics of 201 patients diagnosed or followed up because of sarcoidosis and enrolled to study.

The most frequent extrapulmonary and extracardiac locations of sarcoidosis were: spleen (15.9%), peripheral lymph nodes (11.4%), and liver (6.5%). In 10% of the patients, we diagnosed Ca-P balance disturbances. Only 2 of the patients had been treated with corticosteroids in the past (both cases because of pulmonary involvement, several years previously).

Cardiac involvement corresponding with active myocarditis owing to sarcoidosis in CMR was found in 49 patients (24.4%). In 3 cases, viral myocarditis was serologically confirmed (not recognized as CS). In 3 other patients, angiography was done to rule out coronary stenosis as the cause of the observed changes. Tables 2 and 3 show the differences and the distribution of abnormal results of chosen diagnostic tests between the CS-positive and negative groups according to CMR study results.

**Table 2:**
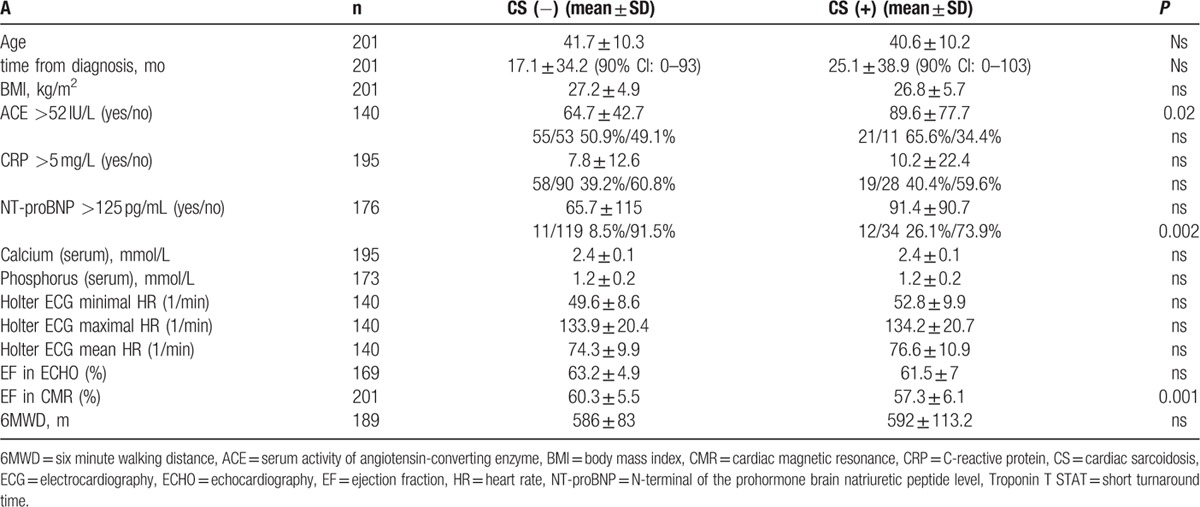
The differences between the CS-positive and -negative groups according to results of CMR study.

**Table 3:**
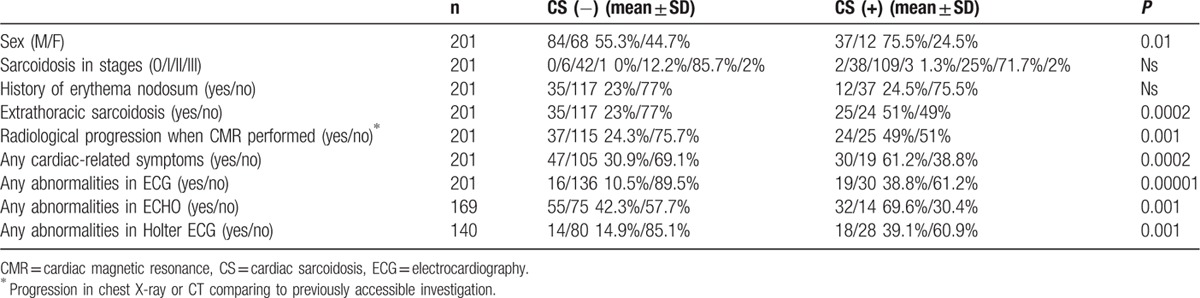
Distribution of normal and abnormal tests results and data from history in CS-positive and -negative groups.

Regarding the results of CMR study as a “gold standard” method, we calculated the sensitivity and specificity, as well negative and positive predictive values for symptoms and laboratory tests suggesting cardiac involvement (Table 4 for details).

**Table 4:**
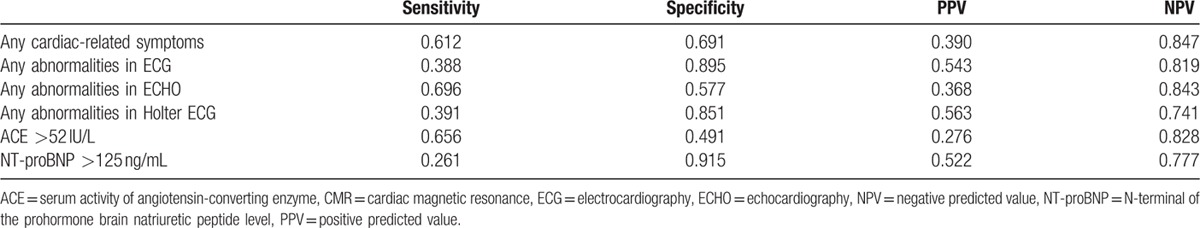
Sensitivity, specificity, PPV, and NPV for commonly used indicators of cardiac sarcoidosis (CMR study results as a reference method).

Analysis of differences between CS-positive and -negative group suggested some features as potential factors increasing chance for CS. When analyzed separately (univariate analysis), the odds ratio (OR) for having cardiac involvement in men compared to women was 2.5. Symptoms suggesting CS were present in 61% of the patients (OR: 3.5). Symptom rates in CS-positive and negative groups are presented in Table 5.

**Table 5:**
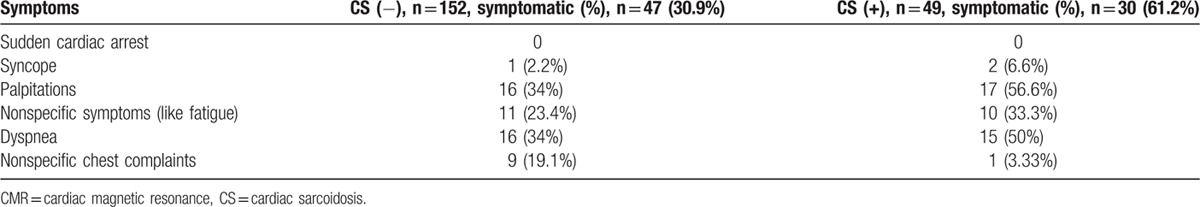
Symptoms reported by patients in CS (+) and CS (−)groups according to the results of CMR study.

**Figure 1:**
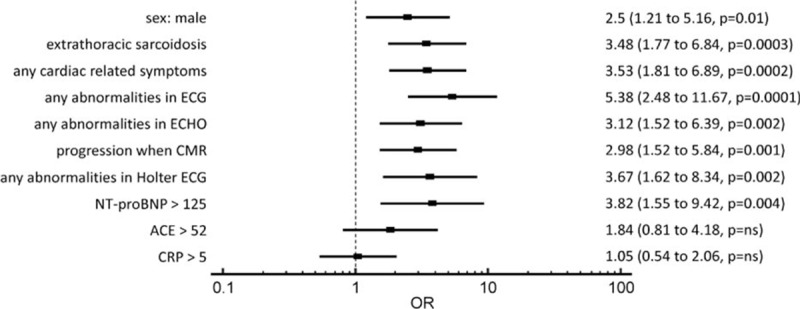
Forest plot: odds ratios (ORs) of identified risk factors for cardiac sarcoidosis. ACE >52 = serum activity of angiotensin-converting enzyme >52 IU/L, CMR = cardiac magnetic resonance, CRP >5 = C-reactive protein >5 mg/L, ECG = electrocardiography, ECHO = echocardiography, NT-proBNP >125 = N-terminal of the prohormone brain natriuretic peptide level >125 pg/mL.

Extrathoracic sarcoidosis also was a risk factor for CS (OR: 3.5). In 24 (49%) cases, CS was diagnosed contemporary with radiological progression in the lungs (OR: 3.0). Elevated serum NT-proBNP (>125) was also identified as increasing the chance for CS (OR: 3.8). Another factor associated with cardiac involvement was any changes in routine electrocardiography (OR: 5.4). Elevated C-reactive protein (CRP) and serum ACE were not associated with an increased risk of CS. The forest plot for mentioned risk factors with detailed numeric data is presented in Figure 1.

We performed logistic regression analysis in 176 patients in whom results of all tests were accessible. The regression equation had the form:

y = −3.75 + 1.53 × (sex: male = 1; female = 0) + 1.05 × (symptoms: yes = 1; no = 0) + 1.17 × (ECG changes: yes = 1; no = 0) + 1.00 × (extrathoracic sarcoidosis: yes = 1; no = 0) + 0.96 × (progressive disease: yes = 1; no = 0) + 1.56 × (NT-proBNP >125: yes = 1; no = 0).

Taking into account the risk factors for CS and analyzing their significance in multivariate analysis, we developed a scoring system (CS Risk Index [CSRI]) setting arbitrary points (but in some proportion to coefficients in regression equation) to chosen factors as presented in Table 6.

**Table 6:**
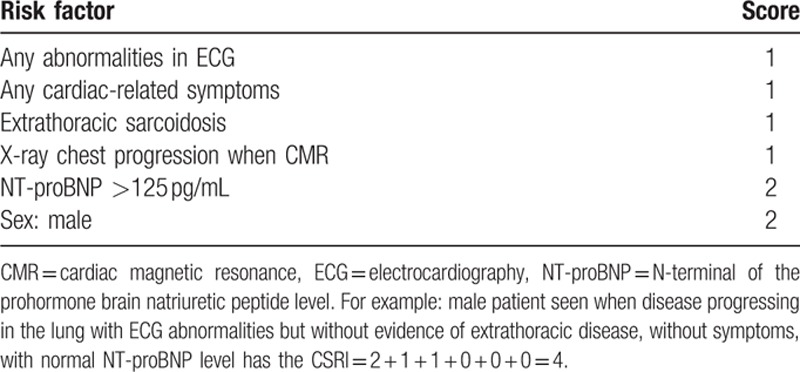
Cardiac sarcoidosis risk index.

For example, a male patient seen when disease progressing in the lung with ECG abnormalities but without evidence of extrathoracic disease, without symptoms, with normal NT-proBNP level has the CSRI = 2 + 1 + 1 + 0 + 0 + 0 = 4. Factors like abnormalities in Holter ECG and echocardiography as well continuous variables (e.g., ACE, CRP, NT-proBNP) were not significant in the multivariate models.

**Figure 2:**
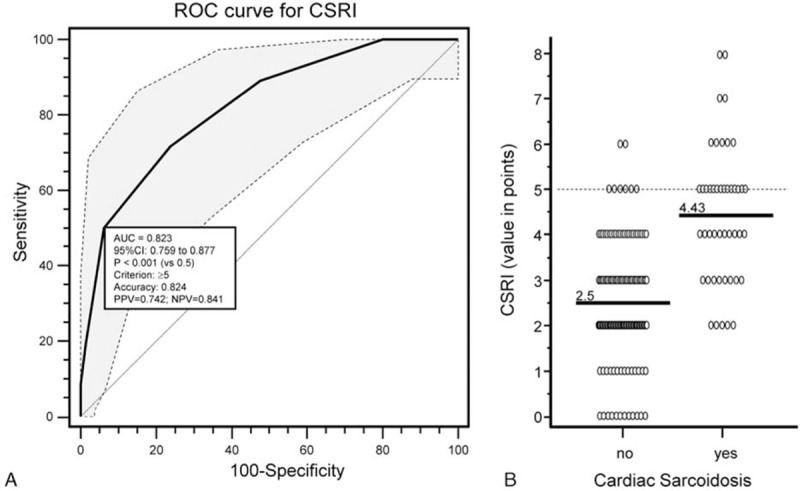
(A) Receiver-operating characteristic (ROC) curve with 95% confidence interval (shadow area between dotted lines) and (B) dots diagram for proposed CSRI, dotted horizontal line at the level of chosen criterion of best accuracy, solid lines at the levels of median values. AUC = area under curve, CSRI = cardiac sarcoidosis risk index.

Using the proposed scoring system, we calculated a score of CSRI for every patient and performed ROC curve analysis (Fig. 2). The mean value of CSRI was significantly different between CS-positive and negative groups (4.43 ± 1.52 vs. 2.5 ± 1.36. respectively, *P* < 0.0001).

The best accuracy was achieved at the level of 5 points with a sensitivity of only 50%, but specificity 94%. The negative predicted value was 0.84 and positive predicted value 0.74 with accuracy 0.82. Detailed data of sensitivity, specificity, and accuracy at given CS risk index levels are presented in eTable 1 (see in 3 Supplemental Digital Content). Table 7 shows the numbers of patients with positive and negative results of CMR study with likelihood ratio in chosen CSRI intervals.

**Table 7:**
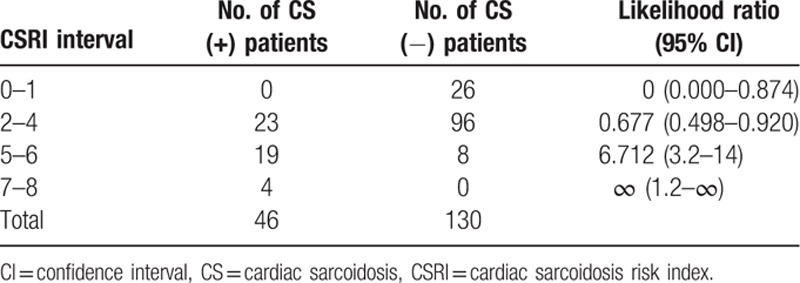
Numbers of CS positive (+) and negative (−) patients according to chosen CSRI intervals with likelihood ratio.

None of 26 patients with a CS risk index of 0 or 1 had CS (all females). At the levels from 2 to 4, the post-test probability of CS (assuming 25% prevalence) was comparable to pretest probability. In the group of patients with the score ≥5, post-test probability increased almost 3 times reaching 70%. All patients with a CSRI 7 and 8 had CS. Only 8 patients without CS had CSRI at the level 5 to 6 and none of them had CSRI 7 or 8.

We also tested different versions of the index with lower numbers of components (e.g., in case of inaccessible NT-proBNP measurement), but these produced significantly lower AUC under ROC curve and accuracy.

## 4 Discussion

This is one of the largest screening studies investigating the incidence of CS using CMRs. CMR with LGE imaging is regarded as a study of choice for diagnosis cardiac involvement in sarcoidosis.[ ^22^ ^27^ ^28^] All 49 diagnosed cardiac sarcoidosis patients fulfilled the criteria of active myocardial inflammation owing to sarcoidosis. ^[26]^ Choosing this criterion, we wanted to avoid erroneous categorization in the case of fibrosis of undetermined etiology. Our results confirm active heart involvement in one-fourth of the patients, a similar percentage as previous autopsy findings. ^[9]^ The incidence is much higher than we found earlier in a symptom-based cross-sectional study (4.7% in the cohort of 1375 sarcoidosis patients). ^[29]^ This study confirms that the prevalence of CS is underestimated unless CMRs are done.

The diagnosis of CS remains difficult. Cardiac involvement may occur at any stage of sarcoidosis and significantly affects the outcome of the disease. An endomyocardial biopsy has a poor sensitivity (about 36%), probably because of the patchy nature of sarcoidosis.[ ^12^ ^30^] That is why this invasive procedure should not be a part of routine evaluation in patients with suspected CS. ^[31]^ The diagnostic criteria were first developed by the Japanese Ministry of Health and Welfare (JMHW) in 1993, modified in 2006. They proposed in the absence of endomyocardial biopsy to identify cardiac involvement by the abnormal function or imaging damages. ^[32]^ The development of imaging techniques has enabled the identification of CS changes at an earlier, less advanced stage of the disease.[ ^17^ ^22^ ^28^ ^33^ ^34^ ^35^] In 2014, (later than we started our study) experts of WASOG (World Association for Sarcoidosis and Other Granulomatous Disorders) and HRS (Heart Rhythm Association) proposed new criteria for the diagnosis of CS.[ ^14^ ^15^] The diagnostic criteria of CS for our study were in line with these proposals.

Recently developed advanced imaging techniques (CMR, PET) are crucial in the recommended pathway for CS diagnosis. However, they may be difficult to obtain in many settings (e.g., routine screening because of their limited availability and cost).

In this screening study, we analyzed factors which may serve as CS predictors. Symptoms are a factor, but are not specific for CS.[ ^36^ ^37^] CS was detected in 39% of our patients with symptoms, indicating that if any symptoms are present, they should not be ignored.[ ^10^ ^21^ ^23^ ^29^ ^38^ ^39^] Unfortunately, the absence of cardiac-related symptoms, despite preserved left ventricular systolic function, does not exclude the diagnosis of CS. ^[24]^

Another interesting result of our study was that males had a higher risk for CS diagnosis than females. Some studies found a higher risks for sarcoidosis for women, whereas others did not.[ ^3^ ^11^ ^13^] A Case Control Etiologic Study of Sarcoidosis (ACCESS), analyzing the large group (of 736 cases) included only newly diagnosed sarcoidosis patients, in whom CS identified in only 17 (2.1%) with no sex predilection.[ ^11^ ^40^] The study of Morimoto et al ^[13]^ indicated that despite more frequent general disease in women, the incidence of heart involvement in both sexes was comparable. The result of our study supports our earlier retrospective investigation performed in a large cohort of 1375 sarcoidosis patients, where men had cardiac involvement significantly more frequently than women (OR 2.34). ^[29]^

The role of the ECG in the diagnosis of CS has been discussed for many years. On the one hand, results of this test could be normal, even in advanced heart involvement, (diagnosed histologically, postmortem). ^[9]^ On the other hand, there is some evidence that ECG abnormalities often occur before the development of cardiac events.[ ^39^ ^41^ ^42^ ^43^] Moreover, ECG is commonly available, an inexpensive tool for practitioners. In our study, any ECG abnormality was associated with a higher risk of CS. Furthermore, we have found that multiorgan and extrathoracic disease (understood as sarcoidosis detected in organs other than lungs, mediastinal lymph nodes and heart) is also predictor for CS. In the retrospective study of Chapelon-Abric et al, ^[10]^ a high rate of neurosarcoidosis was reported. Nagai et al ^[24]^ also noted tendency to more organ involvement in their LGE-positive group. We noted difference between CS-negative and positive patients in serum ACE activity (but small and without difference in distribution of abnormal results), whereas features of active disease, expressed as progression observed in chest X-ray pictures, were related with significantly higher risk of CS. So far, there is lack of an objective, randomized study confirming correlation between radiological pulmonary progression and CS detection; however, one study found an association of abnormal ECG and parenchymal lung infiltrates. ^[39]^

Another marker, which also seems to be a useful tool for identifying CS, is the plasma NT-proBNP level. ^[44]^ In our study, NT-proBNP level was comparable in the CS-negative and positive groups; however, abnormal results (>125 pg/mL) were more frequently detected in CS-positive group (*P* = 0.0023)

Other investigations, performed in this study, like Holter ECG monitoring and ECHO could be also useful as predictors of cardiac involvement, with sensitivity 39% and 70% as well as specificity of 85% and 58%, respectively. In other studies of pulmonary sarcoidosis patients, ECHO abnormalities were described in 14% to 56% of cases.[ ^45^ ^46^] Holter monitoring investigation is superior to conventional ECG for arrhythmias and conduction disturbances detection. In the study of Mehta et al ^[21]^, the presence of abnormal Holter monitoring findings was the most predictive attribute for CS.

We were looking for a simple tool for practitioners, especially working outside large clinical centers, to help in detecting CS. CSRI seems to be a simple tool without need of expensive diagnostic equipment. It may be used as a clinical algorithm in identifying patients that should be more extensively investigated for the presence of CS. The best accuracy of CSRI was achieved at the level of 5 points, where negative predicted value was 0.84. That means that approximately 5 from 6 patients with the scores <5 had a negative CMR investigation. The positive predicted value of 0.74 indicates that 3 of every 4 patients with the scores ≥5 had CS detected. Low CSRI (values 0–1) is possible only in females (as male sex gives 2 points) and is associated with very low probability of CS (none of our patients with such results has CS detected, all were women, 7 of them symptomatic, 4 with radiological progression, 2 with extrathoracic sarcoidosis, and 1 case with ECG changes). Value 2 given only by male sex was present in 31 cases (4 of them CS-positive). Values between 2 and 4 do not change the post-test probability significantly, so should be regarded as uncertain results and are indication for further investigations. CSRI of 7 and 8 was present only in CS-positive patients in investigated group. In our opinion, patients with such high level of CSRI should be urgently diagnosed for heart involvement. However, CSRI of zero does not fully exclude CS. The opinion (and intuition) of the clinician caring for the patient remains important. We encourage lung disease centers to test and evaluate the proposed index.

### 4.1 Limitations of the study

As only one person evaluated the CMR imaging study, interobserver and intraobserver agreement was not assessed (although, unclear, unusual images were assessed by another specialist). Second, we diagnosed CS by CMR study; however, it would be ideal if the diagnosis could be confirmed with CMR-guided endomyocardial biopsy. None of our patients had such a procedure. The proposed CSRI was based only on white population investigated in a single referral center and was not validated in other populations.

## 5 Conclusions

Active cardiac sarcoidosis was diagnosed in 24.4% of our patients with sarcoidosis, much more than in symptoms-based studies. Male sex, cardiac-related symptoms, ECG changes, serum NT-proBNP level, multiorgan involvement, and radiological pulmonary progression taken in a complex may serve as a component for cardiac sarcoidosis risk index. This index may be a good and cost-effective tool for preliminary assessment when access to specialized equipment is limited and helpful for determining the diagnostic urgency.

## Supplementary Material

Supplemental Digital Content
