# Supplementary material for: Prevalence of cardiac sarcoidosis in white population: a case–control study: Proposal for a novel risk index based on commonly available tests
Source: Medicine (Baltimore). 2016 Aug 12;95(32):e4518. doi: 10.1097/MD.0000000000004518 (PMC4985326; doi:10.1097/MD.0000000000004518)
Supplement: Supplemental Digital Content [file medi-95-e4518-s001.doc]

1. **Supplemental Digital Content – eMethods.**

*MM Martusewicz-Boros, PW Boros, E Wiatr, J Zych, D Piotrowska-Kownacka, K Roszkowski-Śliż*

**Title: Prevalence of Cardiac Sarcoidosis in Caucasian Population: A Case-Control Study. Proposal for a Novel Risk Index Based on Commonly Available Tests..**

**eMethods**

The cardiovascular magnetic resonance (CMR) studies were performed using 1.5 T scanner (Siemens, Avanto SQ-engine T-class Tim [76x32]) using a dedicated 32- channel cardiac coil. Study protocol consisted of function, edema and delayed gadolinium enhancement imaging (DE). Left ventricular function was assessed by ECG-gated steady state free precession pulse sequence (SSFP, TrueFISP) in parallel continuous short axis slices. Typical imaging parameters were as follows: echo time (TE) 1,1-1,2 ms, flip angle (FA) 80deg., slice thickness 8 mm, gap 0 mm, GRAPPA 2, matrix 256 x 220, FOV 340-380 mm, 30 phases, temporary resolution ≤45 ms.. Number of slices ranged from 14 to 18 for sufficient coverage of the whole left ventricle from the apex to the mitral valve. Additionally at least one horizontal and one vertical long axis images were obtained for apical segment evaluation.

Black blood T2-weighted short tau inversion recovery (STIR) sequence was used for edema assessment. Sequence parameters were as follows: TE 73 ms, slice thickness 8 mm, ETL 18, inversion time 170 ms for fat suppression, GRAPPA 2, matrix 256 x 192, FOV 300 x 400 mm. Images were obtained every second heartbeat.

DE images were obtained with high resolution IR-prepared gradient echo sequence 15-20 min. after iv. administration of 0,1 mmol/kg Gd-based contrast agent. For DE and T2 weighted STIR imaging the same views and slice thickness were used as for cine imaging; continuous short axis stack, at least one horizontal and vertical long axis. Sequence parameters were as follows: TE minimum, TR minimum, GRAPPA 2, FA 25ͦ , matrix 256 x 220, FOV 340-380 mm. Inversion time (TI) was adapted to null the signal from normal0 myocardium: usually in range of 300-370 ms, based on the scout image results.

The imaging was performed in expiration, during breath holds. Oxygen was supplied during the whole study.

Postprocessing was performed on dedicated workstation (Siemens) using Argus software by a single experienced CMR reader. In some cases the images were supervised by second reader and thus the results was a consensus of both.

Segmental wall motion abnormalities (hipokinesis, akinesis, dyskinesis) were analyzed visually, by reviewing all cine images in cine mode. For quantitative analysis endocardial and epicardial borders were segmented automatically on continuous, parallel short axis slices and then manually corrected. Papillary muscle were included into the cavity volume both at a diastole and systole. For each patient LV end-diastolic volume, end-systolic volume, ejection fraction, stroke volume and mass were calculated.

Only qualitative visual analysis of T2-weighted STIR images was performed in presence of regional increased signal intensity. In other cases myocardial to skeletal muscle signal intensity ratio was calculated. The cut-off SI ratio> 2 was considered with myocardial edema. DE image were evaluated visually for enhancement regions. Presence as well as location of DE were analyzed.

1. **Supplemental Digital Content – eFigure 1.**

*MM Martusewicz-Boros, PW Boros, E Wiatr, J Zych, D Piotrowska-Kownacka, K Roszkowski-Śliż*

**Title: Prevalence of Cardiac Sarcoidosis in Caucasian Population: A Case-Control Study. Proposal for a Novel Risk Index Based on Commonly Available Tests..**

**eFigure 1.**

eFigure 1. An example of positive CMR study.


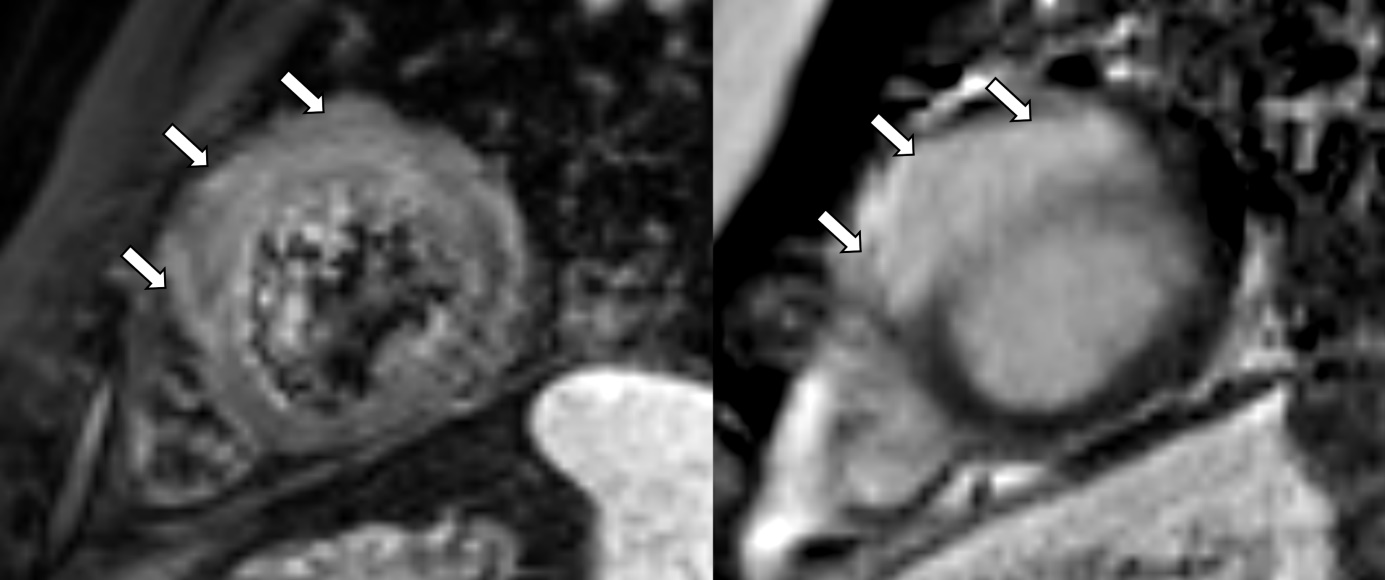


Patient with cardiac sarcoidosis. On the left T2-weighted TSE image with fat saturation in short axis orientation – significantly increased signal in anterior and septal apical segments (arrows). On corresponding Late Gadolinium Enhancement short axis image (on the right) transmural enhancement in anterior and partially septal segments. Thickness of segments with LGE and edema significantly increased.

1. **Supplemental Digital Content – eTable 1.**

*MM Martusewicz-Boros, PW Boros, E Wiatr, J Zych, D Piotrowska-Kownacka, K Roszkowski-Śliż*

**Title: Prevalence of Cardiac Sarcoidosis in Caucasian Population: A Case-Control Study. Proposal for a Novel Risk Index Based on Commonly Available Tests..**

**eTable 1.**

eTable 1. Detailed test data at different levels of CSRI from ROC analysis.

| CSRI | CS(+) | CS(-) | Sensitivity | Specificity | ACC | PPV | NPV | Error rate |
| --- | --- | --- | --- | --- | --- | --- | --- | --- |
| 0 | 0 | 12 | 1.000 | 0.000 | 0.261 | 0.261 |  | 0.74 |
| 1 | 0 | 14 | 1.000 | 0.092 | 0.330 | 0.280 | 1.000 | 0.67 |
| 2 | 5 | 42 | 1.000 | 0.200 | 0.409 | 0.307 | 1.000 | 0.59 |
| 3 | 8 | 31 | 0.891 | 0.523 | 0.619 | 0.398 | 0.932 | 0.38 |
| 4 | 10 | 23 | 0.717 | 0.762 | 0.750 | 0.516 | 0.884 | 0.25 |
| 5 | 14 | 6 | 0.500 | 0.938 | 0.824 | 0.742 | 0.841 | 0.17 |
| 6 | 5 | 2 | 0.196 | 0.985 | 0.778 | 0.818 | 0.776 | 0.22 |
| 7 | 2 | 0 | 0.087 | 1.000 | 0.761 | 1.000 | 0.756 | 0.24 |
| 8 | 2 | 0 | 0.043 | 1.000 | 0.750 | 1.000 | 0.747 | 0.25 |

ROC – Receiver Operating Characteristic; CSRI – Cardiac Sarcoidosis Risk Index; CS - Cardiac Sarcoidosis; (-) – negative, (+) – positive; ACC – Accuracy; PPV – Positive Predicted Value; NPV – Negative Predicted Value
